# Collaborative Penalized Least Squares for Background Correction of Multiple Raman Spectra

**DOI:** 10.1155/2018/9031356

**Published:** 2018-08-29

**Authors:** Long Chen, Yingwen Wu, Tianjun Li, Zhuo Chen

**Affiliations:** ^1^Faculty of Science and Technology, University of Macau, E11 Avenida da Universidade, Taipa, Macau; ^2^Chemistry and Chemical Engineering, College of Biology, Hunan University, Changsha 410082, China

## Abstract

Although Raman spectroscopy has been widely used as a noninvasive analytical tool in various applications, backgrounds in Raman spectra impair its performance in quantitative analysis. Many algorithms have been proposed to separately correct the background spectrum by spectrum. However, in real applications, there are commonly multiple spectra collected from the close locations of a sample or from the same analyte with different concentrations. These spectra are strongly correlated and provide valuable information for more robust background correction. Herein, we propose two new strategies to remove background for a set of related spectra collaboratively. Based on weighted penalized least squares, the new approaches will use the fused weights from multiple spectra or the weights from the average spectrum to estimate the background of each spectrum in the set. Background correction results from both simulated and real experimental data demonstrate that the proposed collaborative approaches outperform traditional algorithms which process spectra individually.

## 1. Introduction

Raman spectroscopy, which provides valuable chemical and physical information of studied samples, is widely used as an analytical tool for many applications like material identification, chemical detection, and biomedical analysis [1–3]. Peaks in Raman spectra are the fingerprints of the analyte, and the corresponding peak heights or peak areas have strong correlations with the concentration of the analyte. However, spectral interferences have a strong negative effect on the measurement of peaks, and this in the long run hinders the performance of Raman spectroscopy-based quantitative analysis [4]. Representative interferences for Raman spectra include backgrounds mainly caused by instrument fluctuations and fluorescent substances. The noises of the instrument and the occasional spikes caused by cosmic rays also deteriorate the quality of Raman spectra. As a result, some preprocessing steps should be conducted to handle the interferences in the Raman spectra. In this paper, we mainly focus on the background correction problem.

Numeric background correction algorithms have been proposed in the past decades for Raman and other spectra. For example, the wavelet transform is used as a powerful tool for background removal by decomposing the Raman signals in the frequency domain [5–7]. Because the performance of wavelet-based approaches is greatly affected by the selection of base wavelets and scales, the adaptive wavelet transform was used in [8] to obtain a multiresolution decomposition of a Raman spectrum. The low-frequency background and high-frequency noise were removed thereafter.

The iterative smoothing algorithms also play an important role in background estimation because the background is usually characterized by its smooth variation. The general procedure of this approach is continually smoothing the spectrum until the background is obtained. Many well-known smoothing filters and their enhancements have been widely used in previous studies for the purpose of iteratively removing peaks and deriving backgrounds in the spectra [9–12]. The drawback of such smoothing algorithms is the difficulty in automating their iterations, although some endeavors have been made on this issue [10, 11].

Curve fitting is to fit the background with appropriate points in the spectrum by some fidelity or loss functions like the least squares [13–15]. Such a kind of selection-then-approximation approach is very similar to the manual background estimation procedure. The simple implementation and short running time of curve fitting have made it one popular background correction method used in real applications. Different curves such as Bezier curves [16], splines [13], and polynomial functions [14, 17] have been used to fit the background. On the contrary, without specifying the curve shape of the background, the penalized least squares- (PLS-) based algorithms attempt to automatically estimate the background by a direct approximation of the spectrum with a penalization on the roughness of the approximated curve [15, 18]. In the PLS-based algorithms, one critical issue is the weight setting for different points in the spectrum. These weights are used to indicate the contribution of corresponding points to the final curve construction. Many methods have been proposed to this end [19, 20], and some automatic setting techniques are also suggested [21, 22].

Some comparisons on different baseline correction approaches have been conducted [23], and the optimal choice of background removal for the statistical analysis of spectra has been explored [24]. However, by far, there is no single automatic method that can well handle all the spectra universally and be regarded as the best. Recently, more new baseline correction algorithms like the ones based on sparse representation [25] and neural networks [26] have been proposed.

In practical applications of Raman spectroscopy, multiple measurements of a given analyte are normal practices. A set of strongly correlated spectra is then obtained although they may be generated under different environmental conditions and sampling protocols. In the quantitative Raman analysis like the mixture analysis and multivariate calibration, except multiple measurements, multiple spectra derived from the same mixture with different analyte concentrations are also correlated. Due to the varying backgrounds and random noises in the set of related spectra, the clear information like the peak locations in one spectrum may not be significant in another spectrum. How do we collaboratively use the valuable information in a set of related spectra for the purpose of spectrum preprocessing? Foist et al. first noticed this problem and proposed a method to denoise multidimensional spectral data collaboratively [27]. For background correction, few approaches have been proposed by utilizing the common characteristics shared in a set of related spectra [6, 28, 29]. For instance, the multiple spectra baseline correction (MSBC) algorithm designed in [28] assumed that the pairwise differences between the background removed spectra are small and inserted a regularization for this prior to the asymmetric least squares.

In this paper, based on PLS, we propose a new approach focusing on collaborative background correction for a set of related spectra. Specifically, our main contribution is to design two ensemble strategies to embed the weight information of PLS from multiple spectra to boost each spectrum's background correction. For PLS, the weight of each point in a spectrum denotes the contribution of the point to the final background estimation. In the first scheme, we directly use the weights derived from the average spectrum (by averaging all the related spectra) to calculate the background of each spectrum. The second scheme applies the average of weights obtained from each spectrum using traditional PLS-based approaches. By using the two schemes, our new approach utilizes the strong correlations among multiple spectra and suppresses the effect of noise and signal variation in different spectra.

To illustrate the advantage of collaboratively calculating the weights for PLS algorithms when handling several related spectra, we combine the adaptive iteratively reweighted penalized least squares algorithm (airPLS) [19] and the morphological weighted penalized least squares algorithm (MPLS) [20] with the proposed weight ensemble strategies. In the experiments on the synthetic and real Raman spectra, these enhanced PLS approaches show accurate and robust background removal capability.

## 2. Theory

### 2.1. PLS for Background Correction

The signal smoothing problem was first proposed by Whittaker in 1922 [30]. The pioneering works on applying PLS for baseline correction were conducted by Eilers et al. more than 10 years ago [31, 32]. The rationale behind PLS is to approximate the observed data by balancing the conflicts between the fidelity to original data and the roughness of fitting data.

Assume that *y* is a vector of the Raman spectrum and *z* is the fitting vector; both of them are with the length of *N* elements. The fitted *z* should keep the fidelity to *y* as well as the roughness of the fitted vector. *F* denotes the fidelity to the Raman spectrum *y*, which can be expressed as the sum of squares of differences between *y* and *z*:

(1) $$\begin{array}{c} F=\overset{N}{\underset{i=1}{\sum }}{\left(y_{i}-z_{i}\right)}^{2}. \end{array}$$

*R* denotes the roughness of the fitting vector *z*, which can be expressed as the sum of squares of differences between each element of *z* and its neighbors:

(2) $$\begin{array}{c} R=\overset{N}{\underset{i=2}{\sum }}{\left(z_{i}-z_{i-1}\right)}^{2}, \end{array}$$

where the square of first differences penalty is adopted in (2) to simplify the presentation. In other cases, it is also a natural way to quantify the roughness by the square of higher-order differences.

The following equation is adopted to measure the balanced combination of fidelity and roughness:

(3) $$\begin{array}{c} Q=F+\lambda R, \end{array}$$

where *λ* is a user adjustable parameter that balances the fidelity and roughness. Larger *λ* favours a smoother fitted vector.

In order to apply the PLS to estimate background, a weight vector *w* was introduced for fidelity; its element *w*_*i*_ can be regarded as a weight that depicts the reliability of point *i* as a part of background. Then, *F* is changed to

(4) $$\begin{array}{c} F=\overset{N}{\underset{i=1}{\sum }}w_{i}{\left(y_{i}-z_{i}\right)}^{2}. \end{array}$$

To solve the minimization problem of (3), we get a linear system by equating the partial derivatives of *Q* to zero (∂*Q*/∂*z*=0), and the matrix form of the obtained linear system is as follows:

(5) $$\begin{array}{c} \left(\mathrm{diag}\left(w\right)+\lambda D^{T}D\right)z=\mathrm{diag}\left(w\right)y, \end{array}$$

where diag(*w*) is a diagonal matrix with *w* on its diagonal and *D* is the derivative of an identity matrix. Finally, we solve the fitting vector as follows:

(6) $$\begin{array}{c} z={\left(\mathrm{diag}\left(w\right)+\lambda D^{T}D\right)}^{-1}\;\mathrm{diag}\left(w\right)y. \end{array}$$

There have been some proposed methods for the weight calculation in PLS. To control the smoothness of the fitted vector iteratively, the airPLS method [19] calculates the weight vector *w* in an adaptive way. *w*_*i*_ in each iteration *t* is obtained as follows:

(7) $$\begin{array}{c} w_{i}=\left\{\begin{array}{cc} 0, & y_{i}\geq z_{i}^{t-1},\\ e^{t\left(y_{i}-z_{i}^{t-1}\right)/\left|d^{t}\right|}, & y_{i}<z_{i}^{t-1}. \end{array}\right. \end{array}$$

The vector *d* ^*t*^ consists of negative elements obtained from the subtraction between *y* and *z* ^*t*−1^ in the *t*th iteration step. The fitted vector *z* ^*t*−1^ in the previous *t* − 1 iteration step is a candidate of the baseline. If the value of the signal *y*_*i*_ is greater than the candidate, it can be seen as a part of the peak, of which the weight is set to zero. If not, the weight is calculated as (7). When the iteration count reaches the maximum or when the following termination criterion is satisfied, the iteration will stop and the final weight vector is used for PLS to generate the background:

(8) $$\begin{array}{c} \left|d^{t}\right|<0.001\times \left|y\right|. \end{array}$$

Unlike airPLS which adjusts the weights adaptively, the MPLS method [20] directly calculates the weight vector by applying the mathematical morphology operations on the spectrum to remove the peaks and generate a rough background firstly. The morphology operation involves an object spectrum *y* and a plane structuring element *E*. The transformation is an opening operation which consists of dilation and erosion. To refine the background, the local minimum points between peak areas are selected as meaningful background points with weight 1, and the remaining points are set with a weight of 0. The weighted PLS is then applied to get the final background.

### 2.2. Collaborative Weighted Penalized Least Squares for Multiple Spectra

As discussed in Introduction, in practical applications of Raman spectroscopy, we may collect strongly related spectra that are from either the same kind of material or the solution with different proportional concentrations. In these cases, we can collaboratively estimate each spectrum's background by comprehensively considering the valuable information shared in the whole set of spectra. Specifically, for the weighted PLS-based approaches, we design two schemes to utilize the global information in the set of spectra for the weight calculation.

The simplest information fusion approach for a set of highly related spectra is to average them. More formally, given a set of *m* spectra *y* ^1^, *y* ^2^, …, *y* ^*m*^, we calculate the average spectrum as follows:

(9) $$\begin{array}{c} y^{\text{avg}}=\overset{m}{\underset{i=1}{\sum }}y^{i}. \end{array}$$

By averaging, the effect of noise on spectra is suppressed, and the average spectrum can be regarded an informative representation of the set of related spectra. With the average spectrum, we can apply some traditional weighed PLS-based approaches to calculate the weights of different points that denote the reliabilities of their reliabilities as some parts of background. Then, the obtained weight vector *w* ^avg^ is used for each single spectrum's background removal. For a more accurate fusion, we may set weights for different spectra in the summation of (9). For example, the high-quality spectrum with a higher signal-noise ratio may take a higher weight to contribute more to the final average spectrum. But in our experiments, we find the simple summation produces good results as well.

The second scheme to fuse the information from multiple spectra is to ensemble the weight vectors of all the spectra. For the spectrum *y* ^*i*^, we first use some traditional weighted PLS-based approaches to calculate the weight vector *w* ^*i*^ for it. Then, the weight vectors for all the related spectra are combined into one as follows:

(10) $$\begin{array}{c} w^{\text{com}}=\overset{m}{\underset{i=1}{\sum }}w^{i}, \end{array}$$

where the combined weight vector *w* ^com^ will be used as the final weight vector in weighted PLS-based background estimation of each spectrum.

To illustrate the application of the two schemes proposed above, we improved the airPLS and MPLS methods by modifying their final weight vectors. In these two PLS-based background estimation methods, no matter the weight vector for points in the spectrum is adaptively adjusted in the iteration (the airPLS case) or the weight vector is directly obtained from morphology operations, the final step to estimate the background is a linear regression step that solves (5) by using (6) with the determined weights.

Given a set of *m* spectra *y* ^1^, *y* ^2^, …, *y* ^*m*^, we have the following 4 enhanced PLS approaches to estimate each spectrum's background.

#### 2.2.1. Average Spectrum-Based airPLS (AS airPLS)

In this method, we first derive the average spectrum *y* ^avg^ from (9). Then, the airPLS is applied over the *y* ^avg^. But here, we only record the weight vector derived at the last iteration of airPLS using (7). This weight vector is denoted as *w* ^avg^. Now, the background of *y* ^*i*^ is calculated by using (6), in which the weight vector *w* is *w* ^avg^ and the spectrum *y* is *y* ^*i*^.

#### 2.2.2. Combined Weight-Based airPLS (CW airPLS)

This method first applies airPLS for each spectrum. We only record the final weight vector for each spectrum obtained in the last iteration of airPLS. Then, the combined weight vector is calculated by using (10), and we use this weight vector to derive each spectrum's background by using (6).

#### 2.2.3. Average Spectrum-Based MPLS (AS MPLS)

This method is very similar to AS airPLS. The only difference is that *w* ^avg^ is calculated by applying MPLS on the average spectrum *y* ^avg^.

#### 2.2.4. Combined Weight-Based MPLS (CW MPLS)

This method is similar to CW airPLS. The difference is that the initial weight vector for each spectrum is obtained by MPLS instead of airPLS.

Because the weight vector is also extensively used in other weighted PLS-based background removal approaches [33–35], including some recently proposed enhancements of airPLS [18], the average spectrum and combined weight-based schemes proposed in this paper can be easily adopted by these approaches to collaboratively process a set of related spectra.

## 3. Experimental

To verify the performance of collaborative approaches proposed in this paper on background removal of a set of related Raman spectra, we compare them with traditional approaches on simulated spectra and real Raman spectra.

### 3.1. Simulated Data

For the simulated data, each spectrum *s* ^*i*^ in the set *S* consists of the pure signal *p* ^*i*^, background *b* ^*i*^, and noise *n* ^*i*^:

(11) $$\begin{array}{c} s^{i}=p^{i}+b^{i}+n^{i}. \end{array}$$

**Figure 1:**
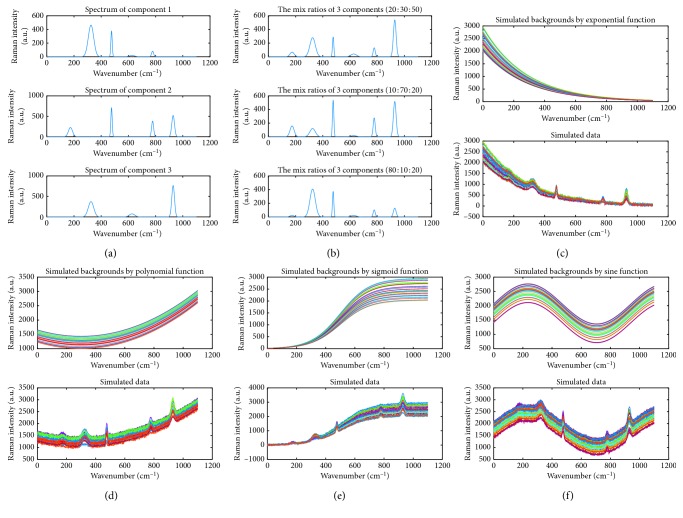
Simulated data. (a) Spectra of three chemical components. (b) Three sampling mixture signals with different concentrations of components. (c–f) The simulated spectra and their backgrounds.

As our methods are to process multiple correlated spectra, the simulated pure signal *p* ^*i*^ is a mixture of three pure spectra illustrated in Figure 1(a) that represent three chemical components. The concentrations of different components are randomly drawn from 0% to 100% in the mixture. By doing this, the simulated pure signals show variations of the peaks.  Figure 1(b) depicts some simulated pure signals with different concentrations of components. In addition, the background *b* ^*i*^ in (11) is generated by exponential, polynomial, sigmoid, or sine curves with a random amplitude. Finally, the Gaussian white noise *n* ^*i*^ is added to the summation of the background and pure signal. Altogether, we randomly generate 30 simulated spectra for each type of backgrounds, as shown in Figures 1(c)–1(f). Their corresponding backgrounds are plotted.

We compare the performance of our improved methods (AS airPLS, AS MPLS, CW airPLS, and CW MPLS) with one of the original airPLS and MPLS methods. Unlike the collaborative AS- and CW-based methods, the airPLS and MPLS process the spectra in the simulated data separately. In addition, the multiple spectra baseline correction (MSBC) algorithm proposed in [28] is also included in comparison.

In these simulated data, as the ground truth backgrounds are known, we can evaluate the performance of each method by calculating the mean squared error (MSE) or the relative error (RE) between the real background *b*_*i*_ and the estimated background ${\tilde{b}}_{i}$:

(12) $$\begin{array}{c} \text{MSE}=\frac{1}{N}\overset{N}{\underset{i=1}{\sum }}{\left(b_{i}-{\tilde{b}}_{i}\right)}^{2}, \end{array}$$

(13) $$\begin{array}{c} \mathrm{Relative}\text{ error}=\frac{1}{N}\overset{N}{\underset{i=1}{\sum }}\frac{\left|b_{i}-{\tilde{b}}_{i}\right|}{b_{i}}\%. \end{array}$$

According to (12) or (13), for each spectrum in the 30 simulated spectra, we can get one MSE or RE value. For the multispectra, we compare the average MSE or average RE of the 30 simulated spectra. The smaller value of MSE or RE denotes the estimated background is more similar to the ground truth. Therefore, the smaller MSE or RE indicates the better background correction method.

**Figure 2:**
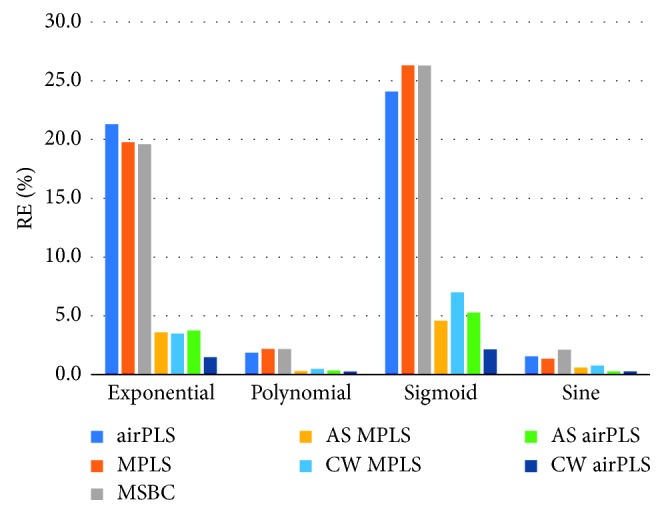
Relative error of different methods on different backgrounds.

As shown in Table 1, our AS- and CW-based methods can obtain much better RE than the traditional approaches. The bar plot in Figure 2 visualizes the results in Table 1. From these results, we can see the multiple spectra baseline correction (MSBC) algorithm performs similar to or a little better than the approaches relying on separate spectrum processing. But its performance is worse than the collaborative approaches proposed in this paper.

**Figure 3:**
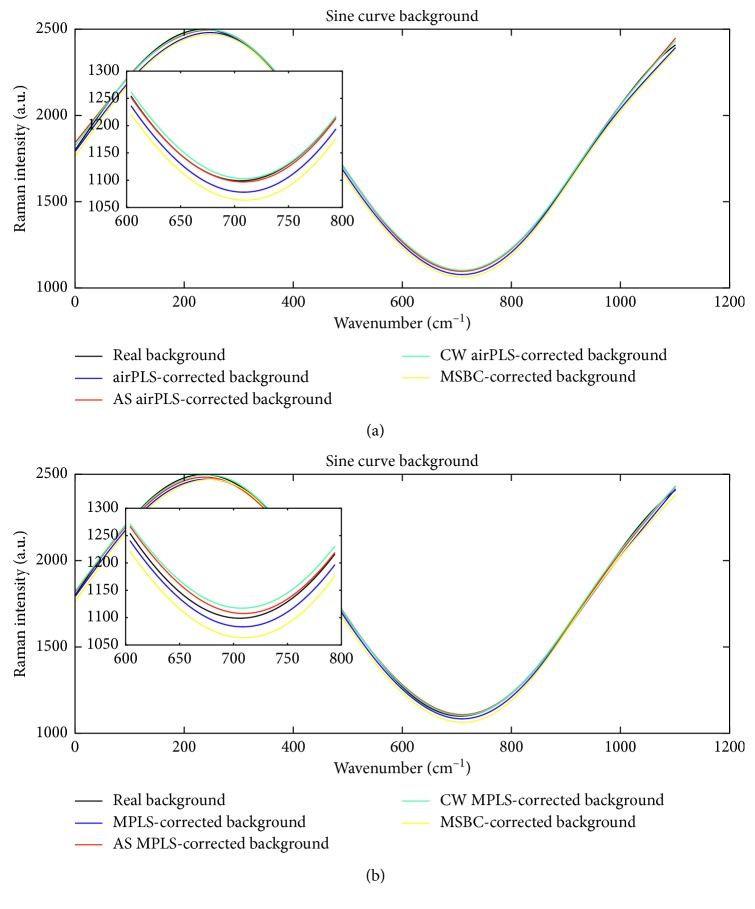
(a, b) Comparison of real backgrounds and estimated backgrounds obtained by different methods.

To have a better comparison, we also plot several representative pairs of ground truth backgrounds (the simulated sine curves) and the estimated ones obtained by different methods in Figure 3. The zoomed-in inserts from 600 cm^−1^ to 800 cm^−1^ are provided in Figure 3 for better visualization. It is pretty obvious that the estimated backgrounds obtained from our enhanced approaches (the red and green lines) are much closer to the real background (the black line).

Because the simulated data are spectra of some mixtures of three chemical components, we can conduct a regression analysis on the preprocessed spectra to predict the concentration values of different components. Here, the principal component regression (PCR) is applied on 29 simulated spectra, and the concentrations of three components in the remainder spectrum are predicted thereafter. We do this 30 times by choosing each simulated spectrum as the testing one once. The root mean squared error (RMSE) is used as the prediction error to evaluate different methods' performance. As shown in Table 2, the spectra preprocessed by the enhanced PLS show higher prediction accuracy when compared with other approaches.

**Figure 4:**
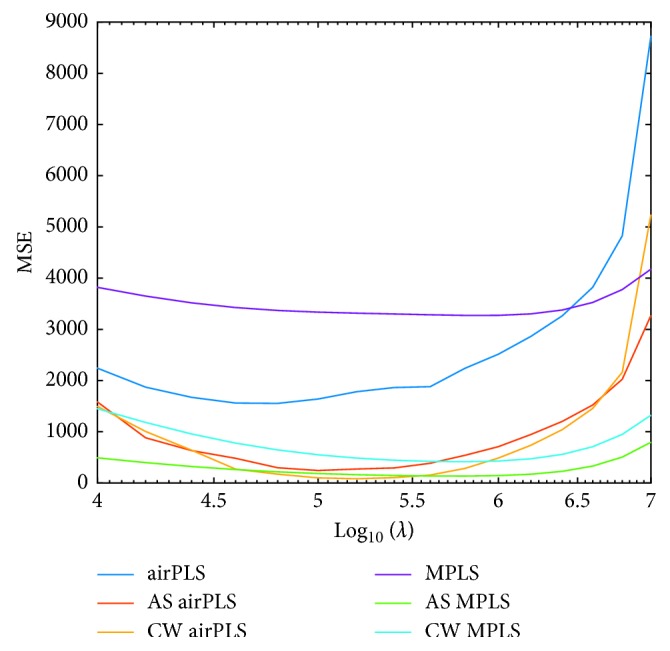
MSE of each PLS method for different *λ*.

For all the six PLS methods, the smoothness parameter *λ* should be adjusted to get a good estimation of background. If *λ* is too small, the estimated background would fit the peaks too much. However, if *λ* is too large, the estimated background would be too smooth to catch the fluctuation of backgrounds. So, it is necessary to delve further into the value of *λ*. As we have mentioned before, we compare the performance of different methods by calculating the MSE between real and estimated backgrounds. Here, we design the experiments with various *λ* and compare the MSE so as to find the optimum *λ*. *λ* is varied in the log scale as is recommended in Eilers' paper [31]. Therefore, we test with *λ* values from 10^2^ to 10^8^ and discard the *λ* value with bad estimated background at first glance. Through comprehensive consideration, we let *λ* to change from 10^4^ to 10^7^ and record the MSE value for each method. The MSE results of each method with various *λ* are displayed in Figure 4. Overall, our collaborative methods can always obtain lower MSE compared with original methods, which means that these methods can get a better estimation of background. The optimal results appear around *λ*=10^5.2^, and similar performance can be obtained between 10^4.8^ and 10^5.8^. It seems that our methods are not too sensitive to *λ*. What it means is that our methods are relatively robust to the choice of *λ*.

### 3.2. Real Raman Spectra

**Figure 5:**
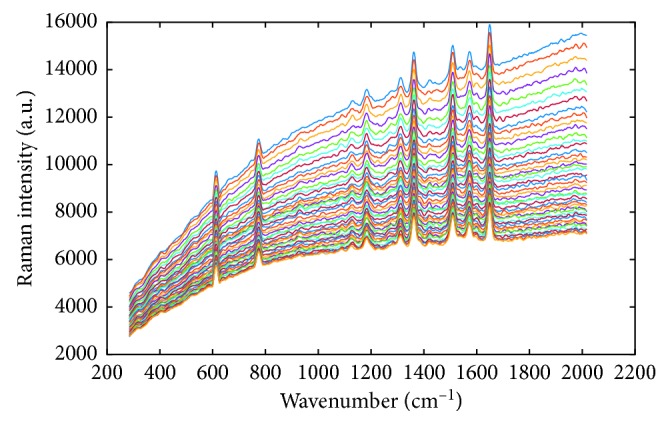
A set of Raman spectra for GIANs collected in a short time.

**Figure 6:**
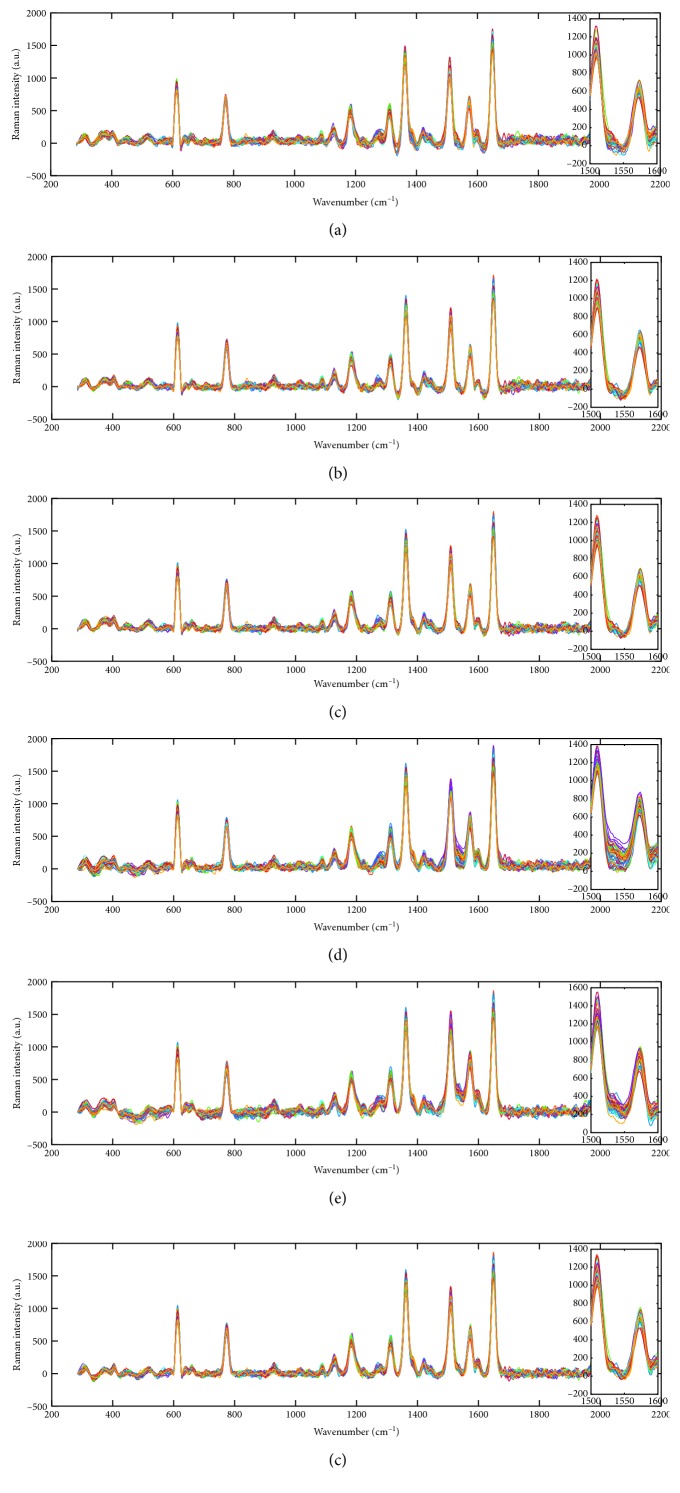
Spectra of GIAN with background removed by airPLS (a), AS airPLS (b), CW airPLS (c), MPLS (d), AS MPLS (e), and CW MPLS (f).

Graphene-isolated-Au-nanocrystal (GIAN) is a unique and stable nanostructure and has been utilized for different biomedical applications, such as sensitive Raman imaging, drug loading for chemotherapy, and photothermal therapy [36]. Herein, we utilized the GIAN to verify the efficiency of proposed background correction approaches. The Raman spectra of GIANs were collected via a static scan in the region of 200–2200 cm^−1^. The spectrum was saved every 0.01 second, and 45 numbers of spectra signals were collected as shown in Figure 5. Because the set of spectra is collection from the same sample in a short time, although the varying backgrounds make the spectra show different intensities, the background-removed spectra should show the same intensity ideally. As shown in Figure 6, the processed spectra by AS- and CW-enhanced PLS methods show more homogeneous features of spectra. This is especially observable in the zoomed region from 1500 cm^−1^ to 1600 cm^−1^.

To quantitatively measure the consistency of background-removed spectra, the average intensity variance of all the processed spectra is calculated. A smaller variance means a closer relation of the background-removed spectra, which indicates a relative better background correction result. From Table 3, we see the variances of the AS and CW methods are smaller than those of the original methods, which implies the better performance of collaborative methods.

**Figure 7:**
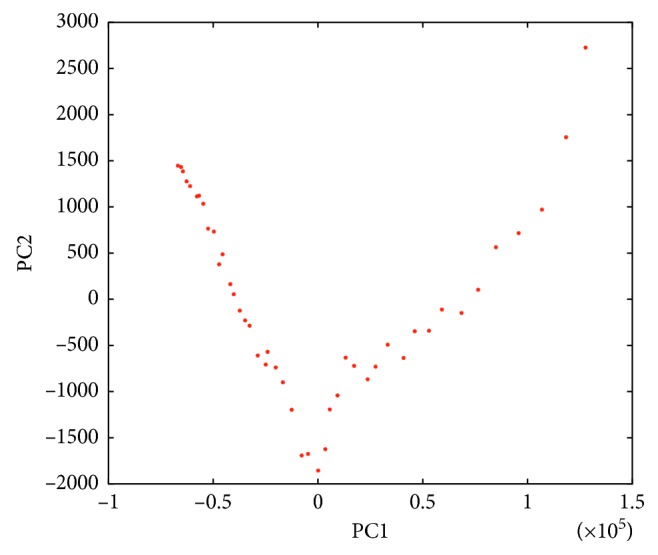
Score plot for original spectra.

**Figure 8:**
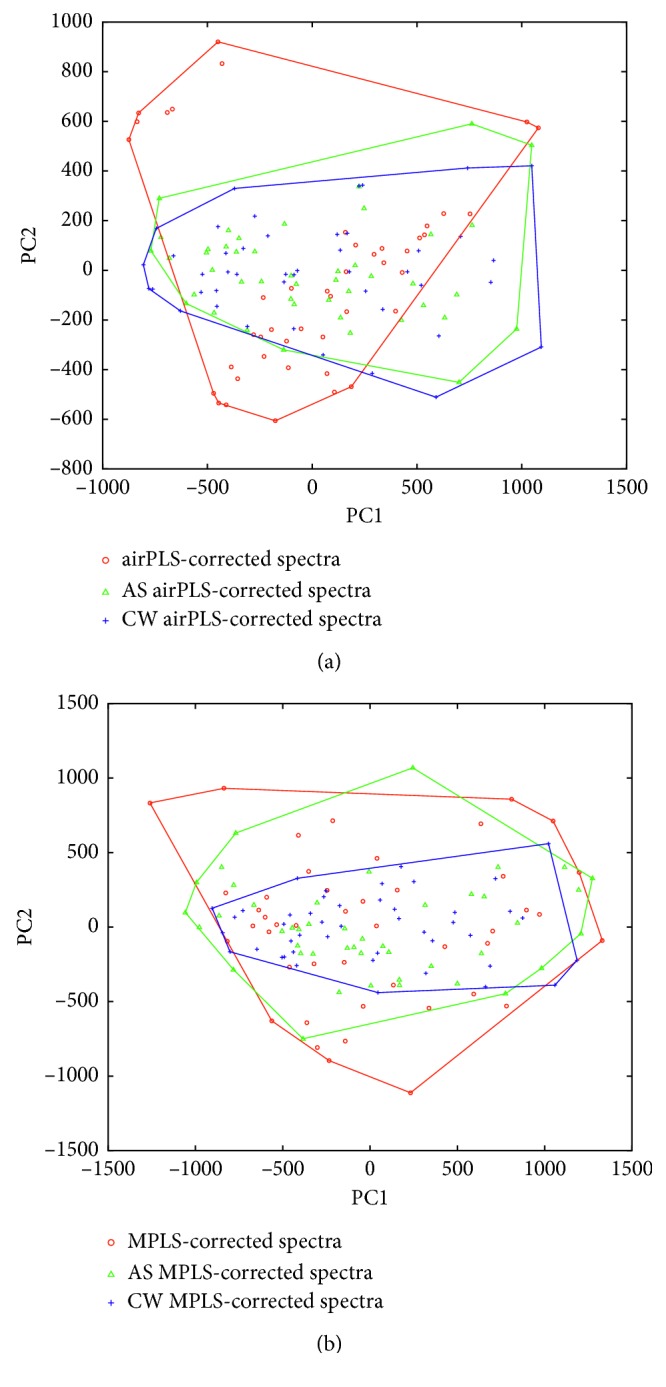
(a, b) Score plots for corrected spectra.

As a commonly used approach to quantify the background correction [19, 20, 35], principal component analysis (PCA) is also conducted on the matrix of spectra before and after background removal. In PCA, the score of a data point along with a principal component (PC) is the distance from the origin to the data point's projection on this PC.  Figure 7 shows the score plot of the original spectra according to the first two principal components. The PCA score plots of background-corrected spectra are shown in Figure 8. For the real Raman data used in this experiment, the discrepancy in spectra is mainly caused by the baseline interference. The effective removal of baselines should increase similarity in processed spectra, which is reflected by a tighter PCA cluster [16]. In Figure 8, the convex hulls of data are also highlighted in score plots to illustrate the compactness of PCA clusters. Clearly, the enhanced PLS methods show tighter convex hulls, and this confirms their advantages over classical approaches.

## 4. Conclusion

Considering the valuable information in the whole set of related spectra, we propose to remove their backgrounds collaboratively. Based on penalized least squares, we improve the background correction methods for a set of correlated Raman spectra by designing two collaborative weighting schemes for background estimations. The average spectrum (AS) method fuses all the considered spectra into an average one to calculate the weights or contributions of different points to the background. The combined weight (CW) method averages the weights derived from all spectra for background estimation. To illustrate the performance of such collaborative approaches on background correction problems, we apply the AS and CW versions of airPLS and MPLS to process simulated and real Raman spectra. The results demonstrate the collaborative approaches are much better than traditional approaches that process spectra individually.

## Figures and Tables

**Table 1:** Relative error for different backgrounds.

	Exponential (%)	Polynomial (%)	Sigmoid (%)	Sine (%)
airPLS	21.3	1.9	24.1	1.5
MPLS	19.8	2.2	26.3	1.3
MSBC	19.6	2.1	26.3	2.1
AS airPLS	3.7	0.3	5.3	0.3
CW airPLS	1.5	0.2	2.1	0.3
AS MPLS	3.6	0.3	4.6	0.6
CW MPLS	3.5	0.5	7.0	0.8

**Table 2:** RMSE of PCR analysis on preprocessed spectra.

	Exponential	Polynomial	Sigmoid	Sine
airPLS	0.0216	0.0202	0.0206	0.0208
MPLS	0.0226	0.0214	0.0224	0.0208
MSBC	0.0249	0.0248	0.0248	0.0249
AS airPLS	0.0181	0.0185	0.0185	0.0185
CW airPLS	0.0219	0.0188	0.0189	0.0189
AS MPLS	0.0186	0.0180	0.0190	0.0188
CW MPLS	0.0203	0.0196	0.0199	0.0193

**Table 3:** Average intensity variance of background-corrected GIAN data.

Method	Variance
airPLS	1101.02
AS airPLS	**880.67**
CW airPLS	914.54
MPLS	1635.78
AS MPLS	1530.35
CW MPLS	**988.65**

## Data Availability

The Raman spectrum data used to support the findings of this study are available from the corresponding author upon request.

## Supplementary Materials

Supplementary Materials The source code for the proposed methods in this study can be accessed at http://www.cis.umac.mo/cybernetics/backRemoveCode.zip. Please download and upzip the file and check the readme.pdf for more information.
