# Supplementary material for: Collaborative Penalized Least Squares for Background Correction of Multiple Raman Spectra
Source: J Anal Methods Chem. 2018 Aug 29;2018:9031356. doi: 10.1155/2018/9031356 (PMC6136554; doi:10.1155/2018/9031356)
Supplement: Supplementary Materials — The source code for the proposed methods in this study can be accessed at http://www.cis.umac.mo/cybernetics/backRemoveCode.zip. Please download and upzip the file and check the readme.pdf for more information. [file 9031356.f1.zip › 9031356.f1/Readme.pdf]

- This folder includes all the MATLAB scripts for the proposed algorithms:
  1. airPLS.m, the MATLAB script of airPLS algorithm for background correction.
  2. MPLS.m, the MATLAB script of MPLS algorithm for background correction.
  3. Morphology.m, the MATLAB script of morphology function in MPLS.
  4. WhittakerSmooth.m, the MATLAB script of weighted smoothing with a finite difference penalty
  5. screan2png.m, the MATLAB script for generating a png file of the current figure.
  6. AS_airPLS.m, the MATLAB script of AS airPLS algorithm proposed in this paper for collaborative spectra background correction.
  7. CW_airPLS.m, the MATLAB script of AS airPLS algorithm proposed in this paper for collaborative spectra background correction.
  8. AS_MPLS.m, the MATLAB script of AS airPLS algorithm proposed in this paper for collaborative spectra background correction.
  9. CW_MPLs.m, the MATLAB script of AS airPLS algorithm proposed in this paper for collaborative spectra background correction.
  10. simulated_compare.m, the main MATLAB script to compare different algorithms.
- To run all the algorithms on the simulated data, please run **simulated_compare.m** in Matlab
- For comparison, the source codes of MPLS and airPLS are included.
- We acknowledge and thank the codes of airPLS and MPLS obtained from Dr. Zhimin Zhang's website.
